# Real-Time Search-Assisted Multiplexed Quantitative Proteomics Reveals System-Wide Translational Regulation of Non-Canonical Short Open Reading Frames

**DOI:** 10.3390/biom13060979

**Published:** 2023-06-12

**Authors:** Hiroko Kozuka-Hata, Tomoko Hiroki, Naoaki Miyamura, Aya Kitamura, Kouhei Tsumoto, Jun-ichiro Inoue, Masaaki Oyama

**Affiliations:** 1 Medical Proteomics Laboratory, The Institute of Medical Science, The University of Tokyo, 4-6-1, Shirokanedai, Minato-ku, Tokyo 108-8639, Japan; 2 Department of Bioengineering, Graduate School of Engineering, The University of Tokyo, 7-3-1, Hongo, Bunkyo-ku, Tokyo 113-8656, Japan; 3 Department of Cancer Biology, The Institute of Medical Science, The University of Tokyo, 4-6-1, Shirokanedai, Minato-ku, Tokyo 108-8639, Japan

**Keywords:** proteomics, real-time search, short open reading frames, histone deacetylase, cancer

## Abstract

Abnormal expression of histone deacetylases (HDACs) is reported to be associated with angiogenesis, metastasis and chemotherapy resistance regarding cancer in a wide range of previous studies. Suberoylanilide hydroxamic acid (SAHA) is well known to function as a pan-inhibitor for HDACs and recognized as one of the therapeutic drug candidates to epigenetically coordinate cancer cell fate regulation on a genomic scale. Here, we established a Real-Time Search (RTS)-assisted mass spectrometric platform for system-wide quantification of translated products encoded by non-canonical short open reading frames (ORFs) as well as already annotated protein coding sequences (CDSs) on the human transciptome and applied this methodology to quantitative proteomic analyses of suberoylanilide hydroxamic acid (SAHA)-treated human HeLa cells to evaluate proteome-wide regulation in response to drug perturbation. Very intriguingly, our RTS-based in-depth proteomic analysis enabled us to identify approximately 5000 novel peptides from the ribosome profiling-based short ORFs encoded in the diversified regions on presumed ‘non-coding’ nucleotide sequences of mRNAs as well as lncRNAs and nonsense mediated decay (NMD) transcripts. Furthermore, TMT-based multiplex large-scale quantification of the whole proteome changes upon differential SAHA treatment unveiled dose-dependent selective translational regulation of a limited fraction of the non-canonical short ORFs in addition to key cell cycle/proliferation-related molecules such as UBE2C, CENPF and PRC1. Our study provided the first system-wide landscape of drug-perturbed translational modulation on both canonical and non-canonical proteome dynamics in human cancer cells.

## 1. Introduction

Abnormal expression of histone deacetylases (HDACs) was known to control cancer cell fate determination in diversified biological contexts such as proliferation, differentiation or apoptosis [1]. Based on the accumulated medical studies regarding cancer, clinical use of HDAC inhibitors has been widely discussed as a promising therapeutic approach against various types of cancers for a long period [2,3]. In 2006, one of the pan-inhibitors for HDACs, suberoylanilide hydroxamic acid (SAHA), was approved by the US Food and Drug Administration (FDA) for the treatment of cutaneous T-cell lymphoma [4]. In addition to the anticancer activity against hematologic cancers, SAHA is also demonstrated to show significant effects on solid tumors through modulation of cell cycle and induction of apoptosis [5,6]. In parallel with human genome sequencing projects, large-scale accumulation of human transcriptome sequences has also been performed and the protein-coding sequence (CDS) on each transcript is defined as the reference protein sequence in datasets such as UniProt [7]. In addition to the curated protein sequence information, high-resolution mass spectrometry-based proteomics has unveiled the existence of translated products from numerous short ORFs which were previously presumed as ‘non-coding’ nucleotide regions based on the conventional rule of translation initiation [8,9,10,11]. Considering that there is increasing evidence of functionally important peptides encoded by previously undefined short ORFs [12,13,14], comprehensive analysis of not only the already defined ‘canonical proteome’ but also short ORF-encoded ‘non-canonical proteome’ needs to be performed for systematic description of drug-induced effects on cancer cells. In this study, we used human HeLa cells as the model cancer cell platform for analyzing SAHA-dependent cellular regulation at the protein expression level [15,16,17]. As a result of high-throughput proteomic detection based on advanced RTS platform on Orbitrap Eclipse Tribrid mass spectrometry instrument [18,19,20], coupled with multi-phase peptide separation through FAIMS Pro ion mobility interface and Vanquish Neo nanoflow liquid-chromatography technology, more than 20,000 peptide sequences were identified not only from the public human protein sequence datasets but also from novel short ORFs previously annotated as potential coding regions by ribosome profiling [21,22]. Very intriguingly, our TMT-based large-scale quantification of the whole proteome changes in response to SAHA treatment unveiled system-wide translational regulation of diversified non-canonical short ORFs in addition to key cell cycle/proliferation-related molecules such as UBE2C, CENPF and PRC1.

## 2. Materials and Methods

### 2.1. Reagents and Antibodies

Anti-Acetyl-Histone H3 (Lys9) (C5B11) Rabbit mAb (9649), Anti-Nestin (10C2) Mouse mAb (33475), Anti-HO-1 (D60G11) Rabbit mAb (5853), Anti-beta-Actin (13E5) Rabbit mAb (4970), Anti-rabbit IgG, HRP-linked Antibody (7074) and Anti-mouse IgG, HRP-linked Antibody (7076) were obtained from Cell Signaling Technology (Danvers, MA, USA). Suberoylanilide hydroxamic acid (SAHA) was purchased from Cayman Chemical (Ann Arbor, MI, USA) and used as dimethylsulfoxide (DMSO) solution.

### 2.2. Cell Culture, Drug Perturbation and TMT Labeling

Human HeLa cells were cultured in Dulbecco’s modified Eagle’s medium (DMEM) media containing 10% fetal bovine serum (FBS) and treated with SAHA for 24 h at discontinuous concentrations of 0, 1, 3, 5 and 10 µM, respectively. Regarding SAHA-dependent perturbation of human HeLa cells, biological replicates were evaluated through Western blots of acetylated Histone H3 in each experiment. The cells were washed three times with PBS, harvested and suspended in 8 M urea containing Benzonase (Novagen, Madison, WI, USA). The cell lysates were quantified using BCA Protein Assay Kit (Thermo Fisher Scientific, Waltham, MA, USA) and 10 µg of the cell lysates treated at different SAHA concentrations was labeled with TMT10plex (Thermo Fisher Scientific, Waltham, MA, USA) according to the manufacturer’s instruction. Briefly, the proteins were reduced with 10 mM tris (2-carboxyethyl) phosphine (TCEP) for 60 min at 56 °C, alkylated with 17 mM iodoacetamide for 30 min. After precipitation with methanol/chloroform, the proteins were reconstituted in 100 mM triethylammonium bicarbonate (TEAB) and digested with Lysyl Endopeptidase, Mass Spectrometry Grade (Fuji Film Wako Chemicals, Osaka, Japan) for 5 h and then with Trypsin Gold, Mass Spectrometry Grade (Promega, Madison, WI, USA) overnight at 37 °C. The fragmented peptide mixtures were then labeled with distinct TMT reagents, mixed and desalted by ZipTip C18 (Millipore, Billerica, MA, USA).

### 2.3. Mass Spectrometry Analysis

Shotgun proteomic analyses were performed by Orbitrap Eclipse Tribrid mass spectrometer with FAIMS Pro interface (Thermo Fisher Scientific, Waltham, MA, USA), which was connected to Vanquish Neo UHPLC system (Thermo Fisher Scientific, Waltham, MA, USA). The peptide samples were separated using a linear gradient of 2–24% mobile phase (0.1% formic acid in acetonitrile) at 300 nL/min. Full-scan MS spectra were acquired with a resolution of 120,000 and subsequent MS/MS scans were performed in the ion trap using collision-induced dissociation (CID) fragmentation with a normalized collision energy of 35% with 10 ms maximum injection time. For the RTS data acquisition on Orbitrap Eclipse Tribrid mass spectrometer, UniProt human reference proteome (UP000005640) database, combined with non-redundant human short ORF sequence data on sORFs.org [22], was used to perform an in-depth identification and quantification of TMT-labeled non-canonical ORFs as well as UniProt-defined protein sequences. In the RTS-MS3 method, MS2 spectra were subjected to RTS using the settings as indicated below; Trypsin was set as enzyme (cleavage next to arginine or lysine, but not before proline). Static modifications were TMT6plex on Lysine (K) and N-Terminus in addition to carbamidomethylation on cysteine (C). Oxidation of methionine (M) was set as a variable modification. Maximum missed cleavages were set to 1 and maximum variable modifications to 2. For TMT-based quantitative proteomic analysis, three technical replicates were measured to calculate log2-transformed fold change and log10-transformed *p*-value, which was adjusted via the Benjamini–Hochberg method.

### 2.4. Protein Identification and Quantification

Protein identification was conducted by searching against the customized database of UniProt human reference proteome (UP000005640) and non-redundant short ORF-encoded human amino acid sequence data extracted from sORFs.org (Table S1) using Sequest HT algorithm in Proteome Discoverer Software (version 2.5) (Thermo Fisher Scientific, Waltham, MA, USA). Cysteine carbamidomethylation and TMT6plex on Lysine (K) and N-Terminus were set as fixed modifications, whereas methionine oxidation and protein N-terminal acetylation were set as variable modifications. A maximum of two missed cleavages was allowed in our database search, while the mass tolerance was set to 10 ppm for peptide masses and 0.6 Da for MS/MS peaks, respectively. In the process of peptide identification, we applied a filter to satisfy a false discovery rate <1%. The obtained quantitative proteomic data was statistically evaluated by Student’s t-test and visualized as volcano plots for respective SAHA concentrations. The *y*-axis represents log10-transformed *p*-value adjusted by Benjamini–Hochberg method, whereas the *x*-axis indicates the log2-transformed fold change of each protein amount in response to SAHA treatment.

### 2.5. Western Blotting Analysis

Human HeLa cells were cultured in DMEM media containing 10% FBS and treated with SAHA for 24 h at different concentrations of 0, 1, 3, 5 and 10 µM in the same manner as the sample preparation for mass spectrometric analysis. After the cells were lysed in the lysis buffer (8 M Urea, 500 mM Tris-HCl, pH 8.2), the lysates were separated on SDS-PAGE and transferred to a PVDF membrane. The membrane was probed with each primary antibody and then with the corresponding HRP-conjugated secondary antibody according to the protocol recommended by the manufacturer of each antibody. The blots were exposed to Clarity^TM^ Western ECL Substrate (BIO-RAD, Hercules, CA, USA) and analyzed by iBright FL1500 imaging system (Thermo Fisher Scientific, Waltham, MA, USA).

## 3. Results and Discussion

### 3.1. Identification and Quantification of SAHA-Regulated Human Proteins Already Defined in the Public Protein Sequence Database

**Figure 1:**
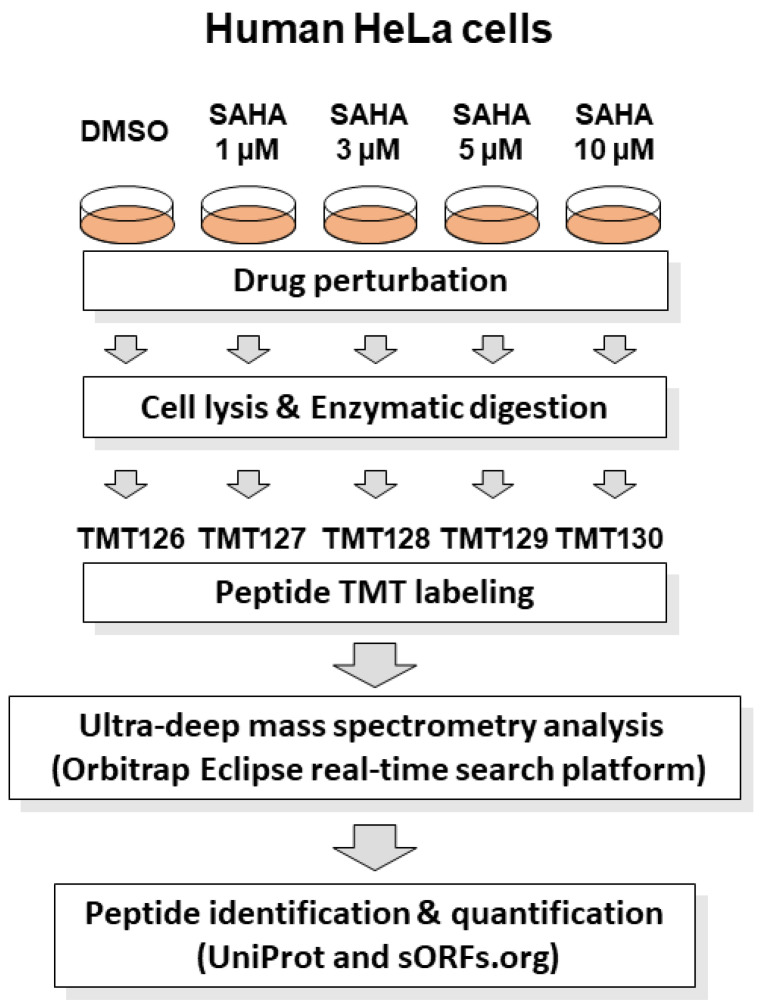
Schematic representation of RTS-dependent in-depth quantitative proteomic analysis of SAHA-treated human cancer cells. The human HeLa cell lysates treated with different concentrations of SAHA (0, 1, 3, 5 and 10 µM) for 24 h were tryptic digested and labeled with discrete TMT tags (TMT126, TMT127, TMT128, TMT129 and TMT130), respectively. The TMT-labeled peptide mixture was subjected to mass spectrometry analysis in an RTS-dependent data acquisition on Orbitrap Eclipse Tribrid mass spectrometer using UniProt human reference protein sequences combined with human short ORF data from sORFs.org [22]. Protein identification and quantification was conducted by searching against the above integrated protein sequence database.

SAHA is reported to show anticancer effects on human HeLa cells according to previous studies [15,16,17]. For system-wide quantitative evaluation of SAHA-induced cellular proteome regulation, we performed a TMT-based proteomic analysis of drug-perturbed human HeLa cells using advanced RTS platform on Orbitrap Eclipse Tribrid MS system. This platform enabled us to greatly improve data acquisition efficiency via an online database search in comparison with conventional post-acquisition data analysis, leading to enhanced quantification of low-abundant peptides. The cell lysates treated with different concentrations of SAHA (0, 1, 3, 5, 10 µM) for 24 h were tryptic digested and labeled with discrete TMT tags as described in Figure 1.

**Figure 2:**
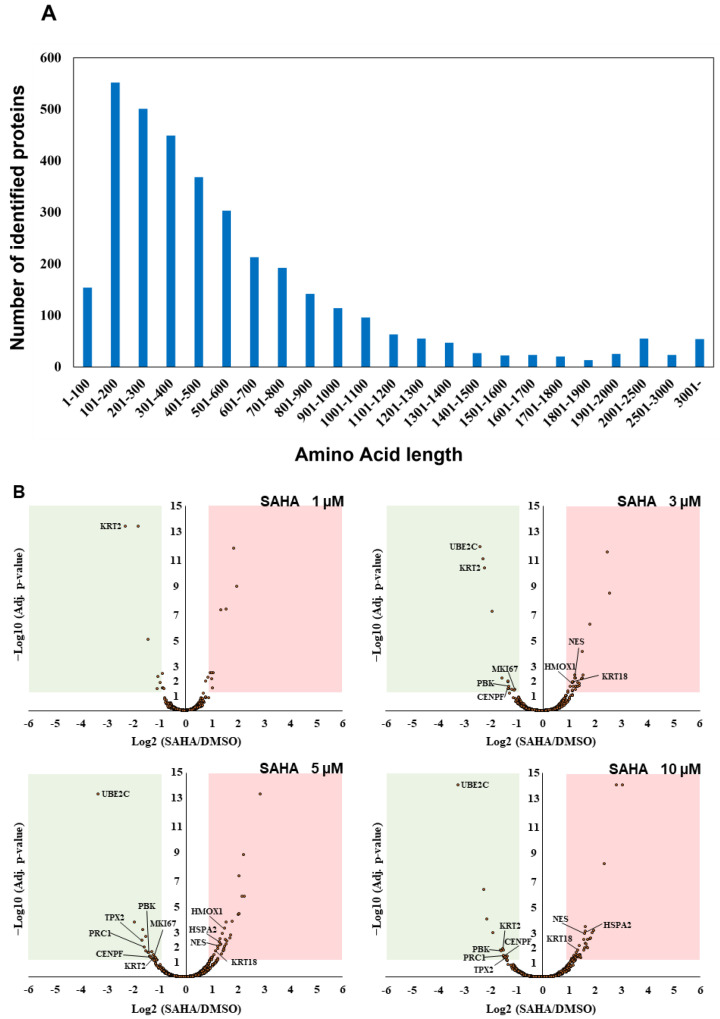

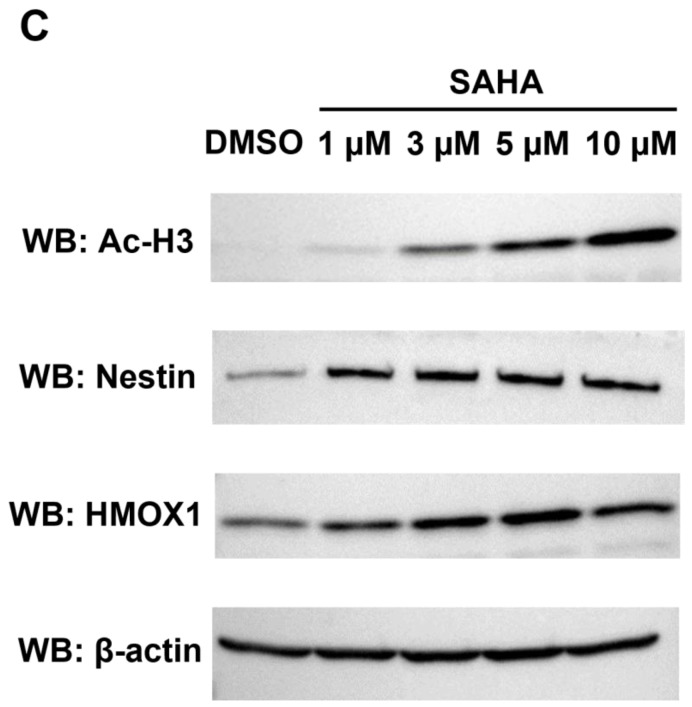
Identification and quantification of UniProt-defined human protein sequences by RTS-assisted shotgun MS analysis. (**A**) Numerical distribution of UniProt-defined human proteins identified in our RTS-assisted proteomic analysis. (**B**) Volcano plots for TMT-based quantitative proteomic changes in response to differential SAHA treatment. The red dots indicate the corresponding data on each quantified protein. The y-axis represents log10-transformed p-value adjusted by Benjamini–Hochberg method, whereas the x-axis indicates the log2-transformed fold change of each protein amount in response to SAHA treatment. (**C**) Western blot analysis of representative cell cycle/proliferation-related proteins upregulated upon SAHA treatment. The acetylation status of histone H3 was also evaluated for validating a dose-dependent effect of SAHA addition on human HeLa cells in each sample set.

Our triplicate mass spectrometric analysis led us to identify 15,664 peptides from UniProt human reference protein sequence database in total (Table S2). The number of the identified short proteins less than 100 amino acids in length was found to be relatively small in our unbiased whole proteome analysis, which reflected the tendency of the distribution of the UniProt human protein sequence datasets (Figure 2A). Furthermore, TMT-based multiplex quantification of four different SAHA-perturbed conditions in comparison with DMSO treatment enabled us to systematically evaluate drug-dependent translational regulation in a statistically stringent manner and revealed that, among 2509 UniProt-defined human proteins quantified, 11 cell cycle/proliferation-related proteins were significantly regulated with more than two-fold change under multiple SAHA conditions examined in this study (Table 1).

Many of the key cell cycle/proliferation-related molecules such as UBE2C, CENPF and PRC1 were found to be decreased in response to SAHA treatment, which was considered to correlate with overall suppression of cancer cell proliferation through cell cycle regulation (Figure 2B, Table S3). In contrast, our quantitative analysis also revealed that, among the other cell cycle/proliferation-related proteins identified, some of the Keap1-Nrf2 signaling molecules such as NES and HMOX1 were inversely increased at the protein level [23], which was validated by Western blot analyses (Figure 2C). These upregulated molecules might be involved in regulating apoptosis under the SAHA conditions applied in our analysis. Although SAHA is considered to function as an epigenetic regulator through enhancement of histone acetylation [1,2], our system-wide quantification revealed that a limited portion of the detected proteome was increased as a result of this drug perturbation. The previous transcriptome analysis of SAHA-regulated HeLa cells indicated that NES was upregulated at the transcription level, whereas HMOX1 were not significantly changed in response to SAHA treatment [24]. Considering that both of the above two proteins were found to be increased in our proteomic measurements (Figure 2B,C), it is very probable that SAHA-induced quantitative control of each translated product in human cancer cells should depend on complex biological mechanisms based on not only epigenetic/transcriptional regulation but also post-translational control including protein degradation.

### 3.2. Proteome-Wide Exploration for Novel SAHA-Regulated Proteins Encoded by the Non-Canonical Short ORFs on the Human Transcriptome

The previous mass spectrometry-based analyses focused on small proteins unveiled the existence of non-canonical short ORF-encoded peptides from a variety of human RNA sequences, such as the sequence regions presumed as the ‘untranslated’ regions (UTRs) of mRNAs or ‘non-coding’ RNAs [8,9,10,11]. In addition, the recent ribosome profiling-based RNA-seq technology greatly contributed to transcriptome-wide annotation of potential short coding regions not only from 5′-UTR or 3′-UTR of the already characterized mRNAs but also from various types of non-coding RNAs such as lncRNAs and nonsense mediated decay (NMD) transcripts [22]. In order to explore for novel SAHA-regulated proteins defined by the human transcriptome, we also performed a Sequest-based database search against the sORFs.org sequence dataset, which is a representative storage of short ORF-encoded amino acid sequence data annotated by ribosome profiling [22].

**Figure 3:**
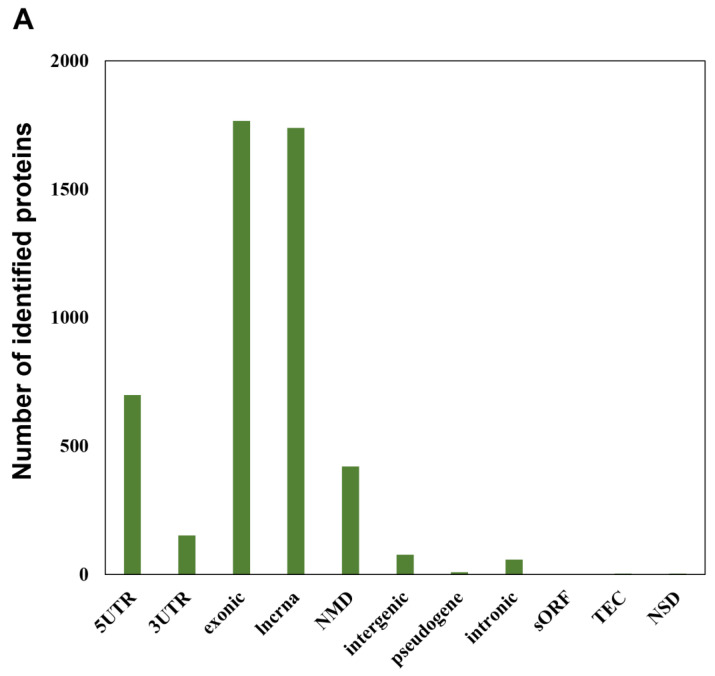

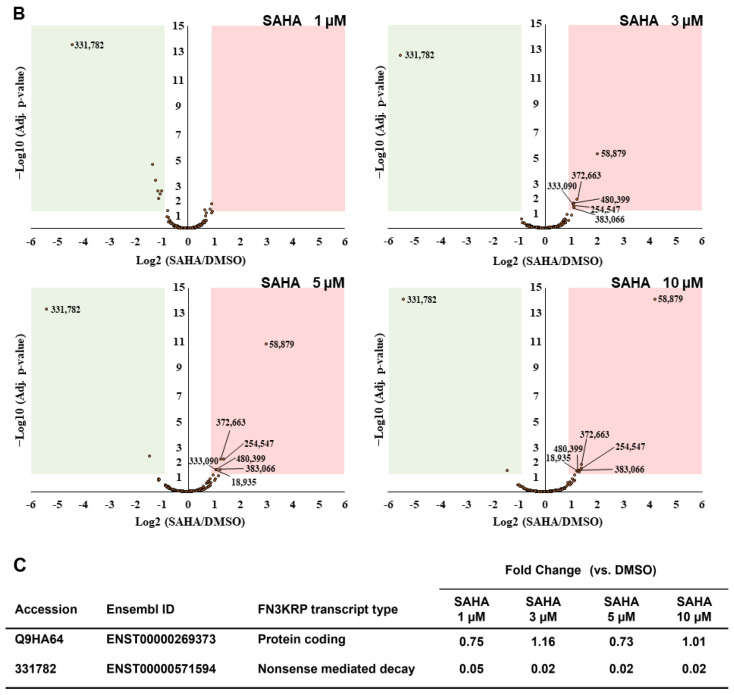
Identification and quantification of non-canonical short ORF-encoded peptides by RTS-assisted shotgun MS analysis. (**A**) Numerical distribution of non-canonical short ORF-encoded peptides identified from the sORFs.org database. The sORF locations classified on each corresponding Ensembl transcript sequence are indicated on the *x*-axis. (**B**) Volcano plots for TMT-based quantitative proteomic changes of non-canonical sORFs in response to differential SAHA treatment. The red dots indicate the corresponding data on each quantified sORF. The y-axis represents log10-transformed p-value adjusted by Benjamini–Hochberg method, whereas the x-axis indicates the log2-transformed fold change of each protein amount in response to SAHA treatment. (**C**) SAHA-dependent differential regulation of the protein products generated from Fructosamine-3-kinase-related protein (FN3KRP) gene locus. Drastic decreased regulation was observed regarding the non-canonical ORF-derived peptide encoded by ENST00000571594, whereas translation of UniProt-defined protein (Q9HA64), which is encoded by the representative transcript (ENST00000269373) on the same gene locus, was not affected by SAHA treatment.

In addition to more than 15,000 peptides derived from UniProt human reference protein sequences as indicated above, our RTS-based in-depth proteomic analysis of SAHA-treated human HeLa cells also unveiled as many as 4997 novel peptide sequences from non-canonical human short ORFs annotated in the sORFs.org database. The detailed sequence information on these non-canonical sORF-encoded peptides indicated that they were mainly encoded by lncRNAs and NMD transcripts as well as the exonic or 5′UTR regions of protein-coding mRNA transcripts (Figure 3A). Our RTS-based highly sensitive proteomic workflow also allowed us to describe SAHA-dependent quantitative profiles of non-canonical short ORF-encoded peptides as well as UniProt-defined proteins and revealed that most of the non-canonical ORF-encoded peptides were unregulated in response to SAHA treatment (Figure 3B, Table S4). Although more than 80% (13,089 out of 15,664 peptides) of the UniProt-derived peptides were successfully quantified to describe dose-dependent regulation of each translated product expressed in human HeLa cells, only less than 10% (427 out of 4997 peptides) of the peptides derived from non-canonical short ORFs were subject to quantification based on their TMT labels, probably due to their low abundance.

Regarding DDR2 and NR1H2, translation of the non-canonical short ORFs encoded in the 5′-UTR region of the corresponding transcripts was found to be significantly upregulated in response to SAHA treatment (Table 2). We also found that the novel short peptides from the lncRNAs defined as ENST00000484265 and ENST00000519322 was translationally enhanced in response to increased SAHA concentrations. Very intriguingly, among 55 NMD transcript-derived peptides quantified, translation of the novel peptide encoded by ENST00000456179, which was transcribed from the FUNDC2 gene locus, were selectively upregulated upon SAHA treatment, whereas the peptide encoded by ENST00000571594 from the FN3KRP gene locus was drastically downregulated at the translational level. Our comprehensive proteomic measurements also unveiled that the amount of the UniProt-defined protein product (Q9HA64) encoded by the alternative transcript (ENST00000269373) from the FN3KRP gene locus was not affected through SAHA-induced perturbation as described in Figure 3C. Further functional analysis of these non-canonical short ORF-encoded regulators would substantially contribute to deepening our understanding of system-wide regulatory effects of SAHA perturbation on human cancer cells.

## 4. Conclusions

The previous mass spectrometry-based integrative proteomic projects led to large-scale identification of novel short peptides derived from a variety of human transcripts previously defined as untranslated regions or non-coding sequences [25,26]. This study has now demonstrated that the ultra-deep quantitative proteomic strategy based on TMT labeling in combination with advanced RTS platform allowed us to evaluate SAHA-dependent translational regulation regarding thousands of non-canonical short ORF-encoded peptides in human cancer cells. Although this RTS-based proteomic approach requires well-curated amino acid sequence data to be prepared from the corresponding transcriptome datasets, our methodology will be widely applicable for evaluating drug-perturbed “hidden proteome” dynamics in every biological context.

## Figures and Tables

**Table 1:** List of the cell cycle/proliferation-related proteins significantly regulated in response to SAHA treatment.

Accession	Gene Symbol	Description	Fold Change (vs. DMSO)			
			SAHA 1 µM	SAHA 3 µM	SAHA 5 µM	SAHA 10 µM
P05783	KRT18	Keratin, type I cytoskeletal 18	1.37	2.25	2.23	2.53
P54652	HSPA2	Heat shock-related 70 kDa protein 2	1.40	1.98	2.48	3.13
P48681	NES	Nestin	1.71	2.37	2.36	3.03
P09601	HMOX1	Heme oxygenase 1	1.60	2.23	2.82	1.80
P46013	MKI67	Proliferation marker protein Ki-67	0.70	0.48	0.44	0.47
P35908	KRT2	Keratin, type II cytoskeletal 2 epidermal	0.20	0.22	0.45	0.35
P49454	CENPF	Centromere protein F	0.83	0.41	0.39	0.36
O43663	PRC1	Protein regulator of cytokinesis 1	0.75	0.51	0.34	0.36
O00762	UBE2C	Ubiquitin-conjugating enzyme E2 C	0.57	0.19	0.10	0.11
Q96KB5	PBK	Lymphokine-activated killer T-cell-originated protein kinase	0.72	0.42	0.38	0.33
Q9ULW0	TPX2	Targeting protein for Xklp2	0.67	0.53	0.32	0.37

The proteins diffentially up/down-regulated with more than two fold changes at multiple SAHA conditions are listed.

**Table 2:** List of the novel sORF-encoded proteins significantly regulated in response to SAHA treatment.

sORF Accession	sORF Location	Ensembl ID	Fold Change (vs. DMSO)				Main CDS Accession (Gene Symbol)
			SAHA 1 µM	SAHA 3 µM	SAHA 5 µM	SAHA 10 µM	
372663	lncrna	ENST00000484265	1.81	2.36	2.42	2.63	
480399	lncrna	ENST00000519322	1.48	2.18	2.16	2.46	
254547	NMD	ENST00000456179	1.59	2.19	2.61	2.68	
383066	5UTR	ENST00000367921	1.60	2.17	2.38	2.62	Q16832 (DDR2)
333090	exonic	ENST00000335661	1.39	2.15	2.14	2.22	Q16548 (BCL2A1)
58879	5UTR	ENST00000599105	1.91	4.05	8.04	18.56	M0R0K3 (NR1H2)
18935	lncrna	ENST00000519322	1.31	1.69	2.13	2.39	
331782	NMD	ENST00000571594	0.05	0.02	0.02	0.02	

The proteins diffentially up/down-regulated with more than two fold changes at multiple SAHA conditions are listed.

## Data Availability

The mass spectrometry proteomics data have been deposited to the ProteomeXchange Consortium via the jPOST repository with the dataset identifier PXD038681.

## Supplementary Materials

The following supporting information can be downloaded at: https://www.mdpi.com/article/10.3390/biom13060979/s1, Table S1: Detailed information on the short ORFs obtained from sORFs.org database; Table S2: TMT-based quantitative proteomics data on SAHA-treated human HeLa cells; Table S3: List of the UniProt-defined proteins regulated in response to differential SAHA treatment; Table S4: List of the novel sORF-encoded proteins regulated in response to differential SAHA treatment.
